# Transtibial pull-out repair of lateral meniscus posterior root is beneficial for graft maturation after anterior cruciate ligament reconstruction: a retrospective study

**DOI:** 10.1186/s12891-022-05406-6

**Published:** 2022-05-12

**Authors:** Mengyuan Li, Zeng Li, Zezhen Li, Hai Jiang, Soomin Lee, Wenhan Huang, Qiujian Zheng

**Affiliations:** 1 Division of Joint Osteopathy and Traumatology, Guangdong Provincial People’s Hospital, Guangdong Academy of Medical Sciences, Guangzhou, 510080 People’s Republic of China; 2 Division of Orthopedics, Guangdong Provincial People’s Hospital, Guangdong Academy of Medical Sciences, Guangzhou, 510080 People’s Republic of China

**Keywords:** Anterior cruciate ligament, Lateral meniscus posterior root, Signal/noise quotient

## Abstract

**Purpose:**

To determine the repair of LMPR lesions would improve the ACL graft maturation.

**Method:**

A total of 49 patients underwent ACL reconstruction were included in this study. Patients were furtherly sub-grouped according to the status of LMPR: intact (17), repair (16) and resected (16). Assessments performed pre- and 2 years post-operatively included patients-reported scores and arthrometer side-to-side difference. Magnetic resonance imaging was used 2 years after the surgery to compare the lateral meniscal extrusion (LME), anterior tibial subluxation of the medial compartment (ATSMC), anterior tibial subluxation of the lateral compartment (ATSLC), the difference of ATSMC and ATSLC, and signal/noise quotient (SNQ) of ACL graft.

**Results:**

In LMPR resected group, it showed greater post-operative ATSMC-ATSLC difference when compared with pre-operatively (*P* = 0.006) and with the other 2 groups (intact: *P* = 0.031; repair: *P* = 0.048). SNQ of ACL graft was higher in LMPR resected group than those in LMPR intact (*P* = 0.004) and repair group (*P* = 0.002). The LMPR repair group showed significant reduction in LME post-operatively (*P* = 0.001). Post-operative measures on ATSLC-ATSMC difference (β = 0.304, *P* = 0.049) and LME (β = 0.492, *P* = 0.003) showed significant association with graft SNQ.

**Conclusions:**

Transtibial repair of LMPR concomitant with ACL reconstruction restored translational stability, reduced meniscus extrusion, making it beneficial for ACL graft maturation.

## Introduction

Meniscus posterior root attachment has been given increasing attention, because tears of this region have been indicated to lead to altered biomechanics in the knee [1–3]. Injuries of posterior meniscus root tears, including radial tears within 1 cm of the meniscal attachment and avulsions of the meniscal attachment, disrupt the hoop tension, and thus lead to meniscal extrusion [4–6]. The peripheral displacement of meniscus has been shown to significantly increase the peak contact pressure in the tibiofemoral compartment, which is biomechanically similar to a total meniscectomy [7]. Besides, the subsequent impairment of wedge effect of posterior meniscus root will furtherly negatively affect the stability and other stabilizer of the knee [8, 9]. Bernholt [10] reported a significantly increased incidence of medial meniscus ramp lesions in patients with LMPRT. According to the current literature, lateral meniscus posterior root (LMPR) tears have been shown to be the concomitant injuries in up to 14% of patients with anterior cruciate ligament (ACL) injuries [11]. Song [12] reported that complete LMPR tear (LMPRT) was associated with high-grade pivot-shift phenomenon in ACL injuries, which indicated the correlation between the LMPR and the subluxation of lateral compartment. Therefore, repair of LMPR has become a hot topic in sports medicine. Currently, transtibial pull-out technique is the most commonly utilized management for LMPRT because this procedure is effective in reducing femorotibial contact area of lateral compartment, improving anterior and rotational stability [3, 13]. According to clinical studies, transtibial pull-out repair of LMPRT improved patient-reported scores, reduced meniscal extrusion and resulted to pleasing healing rate of lateral meniscus based on magnetic resonance imaging (MRI) and second-look arthroscopy [14–17]. However, previous studies mainly focused on the outcome of meniscus rather than ACL graft. There is a lack of research evaluating the ACL graft maturation for those patients with combined ACL reconstruction and LMPR transtibial repair. Whether the repair of LMPRT is associated with the ACL function and maturation has yet to be determined.

The purpose of the present study was to investigate the impact of repaired LMPR on ACL graft function and its mechanical property. We came up with the following hypotheses that the repair of LMPR lesions would improve the prognosis of ACL graft, when using signal/noise quotient (SNQ) under MRI to represent graft mechanical strength and maturation [18].

## Materials and methods

### Study design and setting

This cohort study design was approved by the institutional review board of our institution (KY-Q-2021–278-01) and was performed in strict accordance with the ethical standards stipulated in the 1964 Declaration of Helsinki and its later amendments.

**Fig. 1:**
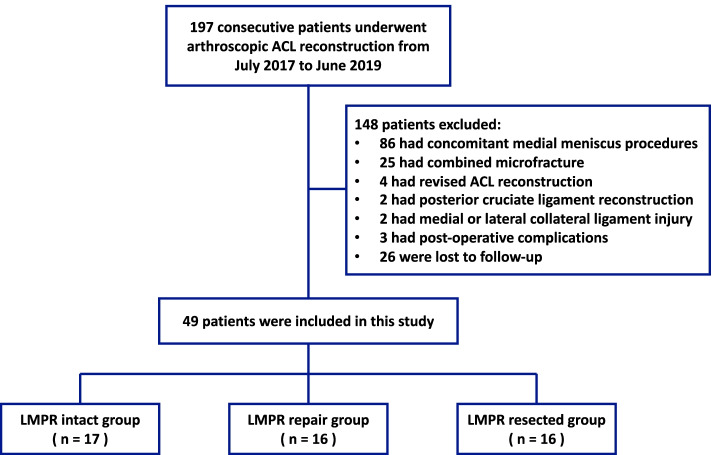
Flowchart of the present study (*ACL* anterior cruciate ligament, *LMPR* lateral meniscus posterior root)

A total of 197 consecutive patients with ACL injuries treated with primary ACL reconstruction in Guangdong Provincial People’s Hospital between July 2017 to June 2019 were retrospectively reviewed. Patients were excluded from this study if they (1) underwent concomitant medial meniscal surgeries, microfracture, previous surgery of affected knee, (2) had posterior cruciate ligament or collateral ligament injury; (3) had significant knee osteoarthritis (Kellgrene-Lawrence grade 3 or 4), (4) had complications, such as infections, deficit in range of motion, re-injury, etc., (5) were not able to have a minimum of 2-year follow-up. Based on this criteria, 49 patients were enrolled in this study (Fig. 1). They were additionally divided into 3 groups. Patients in LMPR intact group received ACL reconstruction without any meniscus procedure, patients in LMPR repair group underwent ACL reconstruction and LMPR repair using transtibial pull-out technique, patients in LMPR resected group had ACL reconstruction and partial meniscectomy for the irreparable lesions of LMPR that progressed to large longitudinal tear, complex tear and large bucket handle tear.

### Surgery

The same experienced surgeon (MYL) performed all the surgeries. The patients were given lumbar anesthesia and then placed in a supine position. Standard diagnostic arthroscopic surgery was performed firstly to confirm ACL injury and concomitant meniscal lesions. The meniscus lesion was repaired or partially resected before ACL reconstruction.

**Fig. 2:**
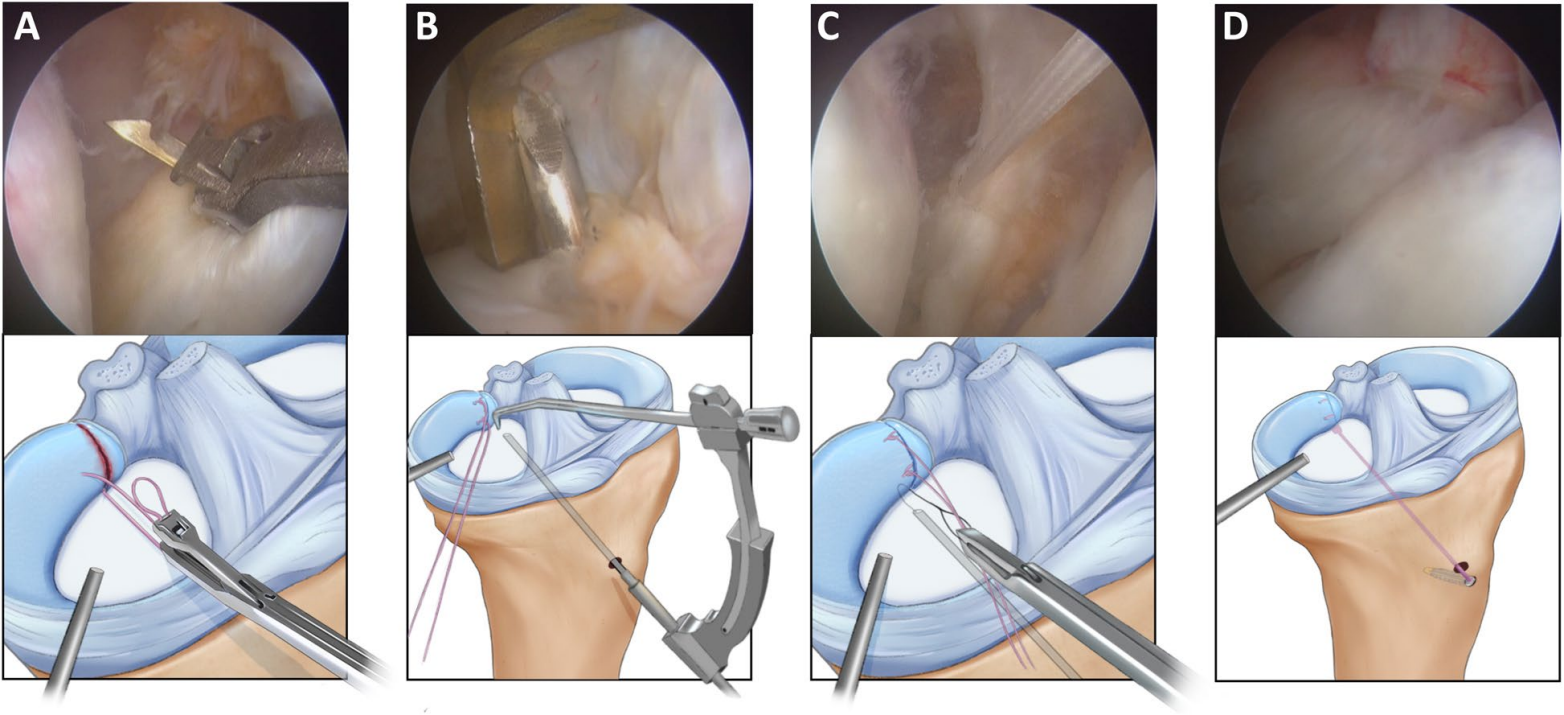
Arthroscopic pull-out repair technique for LMPRT (right knee). **A** A suture was passed through the torn LMPR using a self-retrieving suture passer; **B** A 2.0-mm K-wire was introduced to the tibial attachment of LMPR under the assistance of an ACL tibial guide; **C** A knot was tightened in an all-inside way and then the free ends of the suture were shuttled through the tibial tunnel and the meniscus root was reduced into the tunnel; **D** The tension of the free ends of the suture was adjusted and fixed on the anteromedial tibia using the FootPrint Ultra PK Anchor (Smith & Nephew). (*LMPRT* lateral meniscus posterior root tear, *LMPR* lateral meniscus posterior root, *ACL* anterior cruciate ligament)

For the LMPRT (Fig. 2A), the original tibial attachment site of the LMPR was identified, and then the articular cartilage at the original tibial attachment site was removed using a bur or radiofrequency. A 2–0 TwinFix suture (Smith & Nephew) was passed through the torn LMPR using a self-retrieving suture passer (Arthrex) (Fig. 2B), and then a knot was tightened in an all-inside way (Fig. 2C). An ACL tibial guide (Smith & Nephew) was then introduced through the anteromedial portal with the tip of the guide positioned at the original tibial attachment site of LMPR. A 2.0-mm K-wire was drilled at a 60˚ angle to the tip of the guide. After pulling out the K-wire, an 18-gauge spinal needle with a loop wire was passed through the tibial tunnel to shuttle the free ends of the suture. Finally, the meniscus root was reduced into the tunnel (Fig. 2D), and the tension of the free ends of the suture was adjusted and fixed on the anteromedial tibia using the FootPrint Ultra PK Anchor (Smith & Nephew) before fixing the ACL graft. If the lesion of lateral meniscal horn was irreparable, the injured meniscal tissue was partially resected and the remnant of the posterior horn could be sutured with Fast-Fix device (Smith & Nephew) when possible and necessary.

The hamstring tendons were harvested and knitted as autografts for ACL reconstruction, and anatomic single bundle ACL reconstruction was performed. The femoral tunnel was drilled in the center of anteromedial bundle footprint using femoral tunnel guide (Smith & Nephew) and drilled through the anteromedial portal. The tibial tunnel was drilled with use of tibial tunnel guide (Smith & Nephew) in the center ACL anatomical tibial footprint, which was at the posterior border of the anterior horn of the lateral meniscus. The grafts were fixated with cortical button on femoral side and interference screw on tibial side.

### Rehabilitation

All patients underwent the same postoperative rehabilitation. The elastic bandage was applied immediately after surgery to alleviate pain and knee swelling. Patients were encouraged to perform ankle pump exercise, isometric quadriceps and hamstring contractions, straight and side leg raising exercises the second day. Flexion was restricted between 0˚ and 90˚ during the first 4 weeks postoperatively and gradually advanced as tolerated. Partial weight-bearing exercise was allowed as tolerated at 4 weeks and full weight-bearing was allowed at 8 weeks. Return to sports was allowed gradually with nonpivoting sports at 4 months, but pivoting contact sports were not suggested until 12 months after operation.

### Assessment

Patients’ demographic data including age, sex, body mass index, side of injury, and duration from injury to surgery was collected from the database. Lysholm, Tegner, and International Knee Documentation Committee (IKDC) scores were collected preoperatively and at final follow-up to assess the subjective knee function. A KT-1000 arthrometer (Medmetric) was used to measure the side-to-side difference (SSD) for the assessment of joint stability.

Preoperatively and at final follow-up, a 3.0-T MRI scans (Signa Excite HD; GE Healthcare) for all patients were performed to assess static anterior tibial subluxation of medial and lateral compartment, lateral meniscal extrusion and to measure SNQ of the graft. During examinations, each patient was lying supine in full extension with relaxed quadriceps. The MRI protocols included sagittal, coronal, and axial sequences. Each sequence contained T1- and T2- weighted planes. Slice thickness was 4 mm for each plane.

**Fig. 3:**
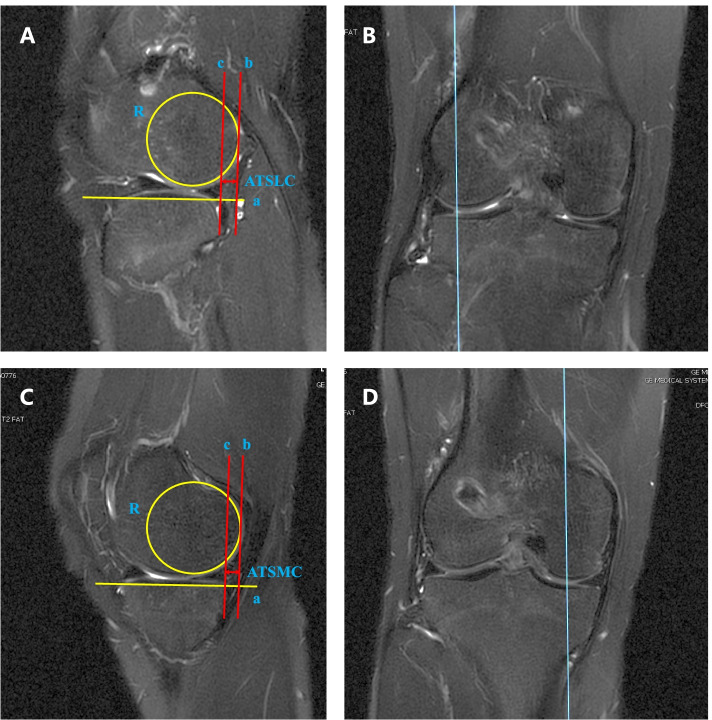
Measurement of ATSLC and ATSMC. **A** For the lateral compartment, the sagittal plane scan that could visualize the most medial image of the fibula at the tibiofibular joint is selected. A best-fit circle (R) over the posterior edge of the lateral femoral condyle was drawn at the subchondral line, and a line tangent to the lateral tibial plateau (a) was also drawn. Then, 2 mutually parallel lines perpendicular to the lateral tibial plateau were determined. The first line (b) crossed the posterior edge of the tibia, and the second line (c) was tangent to the posterior margin of lateral femoral condyle’s best-fit circle. The distance between the two lines was measured as the ATSLC in extension. **B** The coronal image was utilized to help identify the appropriate sagittal plane. **C** A modified method was utilized when measured the ATSMC in extension. **D** The middle aspect of medial condyle was determined when visualizing on the coronal sequences to help identify the appropriate sagittal plane. (*ATSLC* anterior tibial subluxation of lateral compartment, *ATSMC* anterior tibial subluxation of medial compartment)

The relative distance between the posterior edge of femoral condyle and tibial plateau On T2-weighted sagittal sequences was used to assess the anterior tibial subluxation. For the lateral compartment, we selected the sagittal plane scan that could visualize the most medial image of the fibula at the tibiofibular joint. A best-fit circle over the posterior edge of the lateral femoral condyle was drawn at the subchondral line, and a line tangent to the lateral tibial plateau was also drawn. Then, 2 mutually parallel lines perpendicular to the lateral tibial plateau were determined. The first line crossed the posterior edge of the tibia, and the second line was tangent to the posterior margin of lateral femoral condyle’s best-fit circle. The distance between the two lines was measured as the anterior tibial subluxation of lateral compartment (ATSLC) in extension (Fig. 3A, B) [8]. A modified method was utilized when measured the anterior tibial subluxation of medial compartment (ATSMC) in extension. We determined the middle aspect of medial condyle when visualizing on the coronal sequences, and then quantitatively assessed ATSMC on the sagittal image (Fig. 3C, D).

**Fig. 4:**
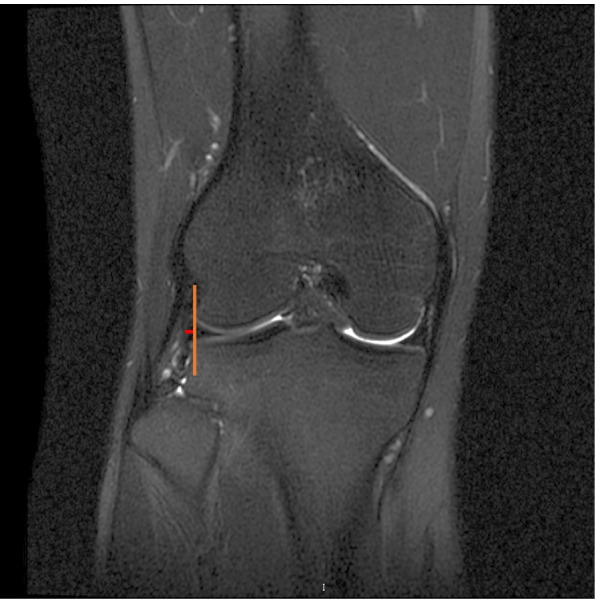
Measurement of LME. Identify a coronal image at the level of the medial collateral ligament, draw a vertical line that indicates the edge of lateral tibial plateau and a horizonal line that is perpendicular to the vertical line. The length of the horizonal line indicates LME. (*LME* lateral meniscus extrusion)

Lateral meniscal extrusion (LME) was measured by the distance from the lateral edge of the tibial plateau to the most lateral aspect of the lateral meniscus on the mid-coronal image by linking the coronal and sagittal image series at the level of the medial collateral ligament (Fig. 4) [19].

**Fig. 5:**
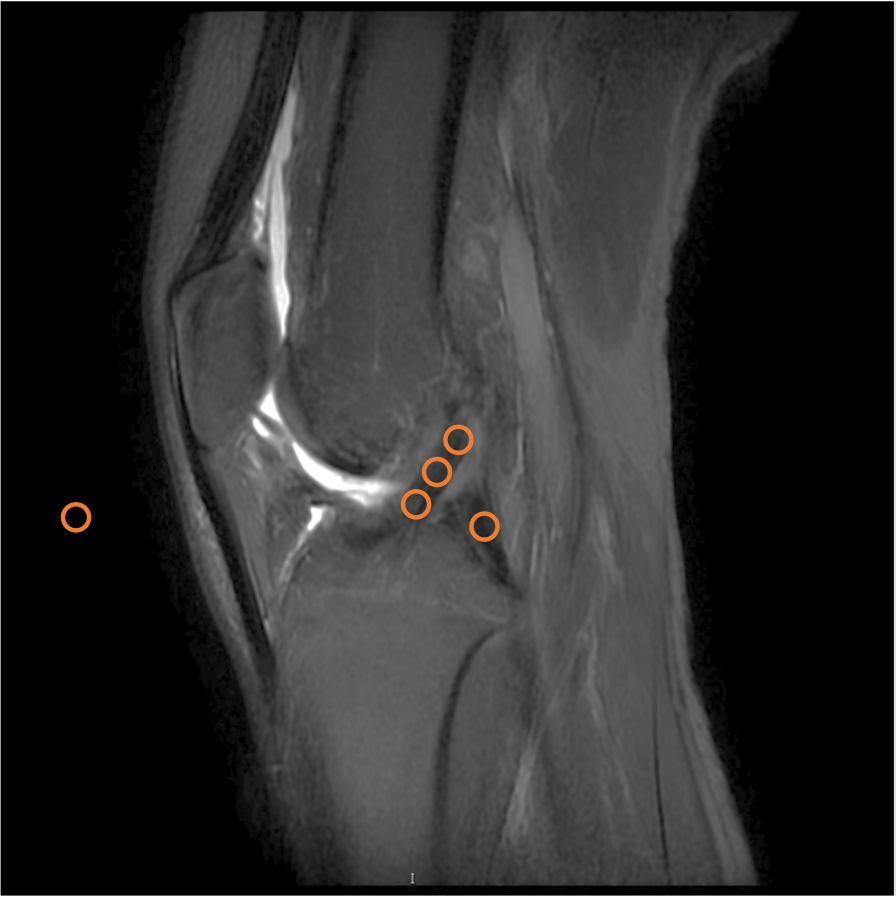
Measurement of SNQ. SNQ for ACL graft was calculated using the formula as followed: SNQ = (graft signal—PCL signal) / background signal. Signal intensity was measured with 15 ~ 20 cm^2^ circular ROIs on T2-weighted sagittal view. The graft signal was measured in its intra-articular portion at superior, middle, and inferior sites in the central slice of ACL. The average was calculated. The signal from the PCL was measured in its distal attachment. The background signal was measured 2 cm anterior to the patellar tendon via the same sagittal image slice. (*SNQ* signal/noise quotient, *ACL* anterior cruciate ligament, *PCL* posterior cruciate ligament, *ROI* regions of interest)

Based on the MRI scans of last follow-up, SNQ for ACL graft was calculated using the formula as followed: SNQ = (graft signal—posterior cruciate ligament [PCL] signal) / background signal. For the MRI analysis, signal intensity was measured with 15 ~ 20 cm^2^ circular regions of interest (ROI) with Picture Archiving and Communication Systems on T2-weighted sagittal view. The graft signal was measured in its intra-articular portion at 3 sites (superior, middle, and inferior) in the central slice of ACL. The average was calculated. The signal from the PCL was measured in its distal attachment. The background signal was measured 2 cm anterior to the patellar tendon via the same sagittal image slice as for graft analysis (Fig. 5) [18, 20]. Healing of the repaired LMPR was classified based on the following criteria: an identifiable meniscus on both sagittal and coronal scans indicated complete healing, absence of an identifiable meniscus or a high signal replacing the normal dark meniscal signal on 1 of the 2 scans indicated partial healing, and no identifiable meniscus on any scans indicated repeated tear [15].

### Statistical analysis

An a priori power analysis was conducted to compute the sample size using the free program, G*Power (University of Dusseldorf, Germany). Based on the previous research of Tang [13], the effect was 0.47. With the power of 0.8 and an alpha value of 0.05, the number of patients required in this study was 16 for each group. The data was described as Means and standard deviations. Chi-square test and Fisher’s exact test were used for categorical variables. Paired-samples T test or Mann–Whitney U test (if data did not meet the assumptions of normality and homoscedasticity) was utilized to compare the variables pre- and post-operatively. The variables between groups were analyzed by 1-way analysis of variance (ANOVA), and the least-significant-difference (LSD) test was performed when significant differences were detected. Univariate and multivariate regression analysis was used to determine which factors influenced maturation of the ACL graft. These statistical analyses were performed using SPSS 26.0 (SPSS Inc, Chicago, IL, USA), the level of statistical significance was set at *P* < 0.05.

## Results

### Demographic and baseline characteristics

A total of 49 patients underwent ACL reconstruction were included in this study, including 17, 16 and 16 in LMPR intact, repair and resected Group, respectively. The duration after the initial onset of knee injury was 15.1 ± 25.5 months, 2.6 ± 2.1 months and 1.5 ± 1.1 month in Group 1, 2, 3 respectively. Demographic characteristics were summarized in Table 1.

**Table 1:** Demographic Data among LMPR intact, repair and resected group

Parameters	LMPR intact (*n* = 17)	LMPR repair (*n* = 16)	LMPR resected (*n* = 16)	*P* value		
				intact vs repair	repair vs resected	resected vs intact
Age, years (mean ± SD)	33.4 ± 9.3	28.0 ± 14.5	36.4 ± 7.6	0.232	0.755	0.139
Gender, male/female, n	11/6	8/7	9/7	intact vs repair vs resected: 0.792		
Height, cm (mean ± SD)	162.3 ± 6.9	167.5 ± 5.3	171.9 ± 7.0	0.773	0.206	0.153
Weight, kg (mean ± SD)	64.0 ± 9.4	63.0 ± 9.0	65.7 ± 11.7	0.801	0.558	0.698
Body mass index, kg/m^2^ (mean ± SD)	22.6 ± 2.8	22.4 ± 2.9	22.1 ± 2.7	0.891	0.793	0.678
Period from injury, months (mean ± SD)	15.1 ± 25.5	2.6 ± 2.1	1.5 ± 1.1	0.084	0.886	0.069
Affected side, left/right, n	9/8	10/6	7/9	intact vs repair vs resected: 0.568		

*SD* standard deviation, *LMPR* lateral meniscus posterior root

### Patient-reported scores

All of the 3 groups showed an improvement in IKDC, Lysholm and Tegner score post-operatively when compared with the pre-operative level. However, LMPR resected group had significantly lower pre- and post-operative Lysholm score than that in LMPR intact and repair group. As far as Tegner score concerned, the significant difference was only detected between LMPR intact and resected group, either pre- or post-operatively (Table 2).

**Table 2:** Patients reported scores and clinical outcomes (mean ± SD) among LMPR intact, repair and resected group

Parameters	LMPR intact	LMPR repair	LMPR resected	*P* value		
				intact vs repair	repair vs resected	resected vs intact
IKDC subjective score						
Pre-operative	59.71 ± 15.98	52.54 ± 19.74	51.78 ± 14.26	0.300	0.920	0.280
Post-operative	89.64 ± 6.31	87.91 ± 13.53	87.67 ± 14.66	0.408	0.917	0.374
* P* value	< 0.001	< 0.001	< 0.001	/	/	/
Lysholm score						
Pre-operative	57.71 ± 18.76	51.27 ± 19.08	33.62 ± 10.39	0.534	0.009	0.001
Post-operative	93.86 ± 14.22	92.54 ± 13.36	88.33 ± 8.53	0.397	0.019	0.002
* P* value	< 0.001	0.001	< 0.001	/	/	/
Tegner score						
Pre-operative	1.50 ± 1.65	0.82 ± 0.75	0.11 ± 0.33	0.156	0.186	0.009
Post-operative	4.21 ± 1.19	3.72 ± 0.47	3.22 ± 0.44	0.162	0.193	0.010
* P* value	< 0.001	< 0.001	< 0.001	/	/	/
Arthrometer SSD, mm						
Pre-operative	5.42 ± 0.62	7.75 ± 0.56	7.57 ± 1.26	< 0.001	0.615	< 0.001
Post-operative	2.06 ± 0.42	2.24 ± 0.32	4.07 ± 0.38	0.229	< 0.001	< 0.001
* P* value	< 0.001	< 0.001	< 0.001	/	/	/

*SD* standard deviation, *LMPR* lateral meniscus posterior root, *IKDC* International Knee Documentation Committee, *SSD* side-to-side difference

### Arthrometer SSD

There existed significant improvements in the arthrometer SSD among the 3 groups post-operatively. In the subgroup analysis, LMPR intact group showed a significantly smaller pre-operative SSD (5.42 ± 0.62 mm) than that in LMPR repair (7.75 ± 0.56 mm, *P* < 0.001) and resected group (7.57 ± 1.26 mm, *P* < 0.001). Nonetheless, after the surgery, the significant greater SSD appeared in LMPR resected (4.07 ± 0.38 mm), while the figure in LMPR repair group (2.24 ± 0.32 mm) reduced to a level equivalent to LMPR intact group (2.06 ± 0.42 mm) (Table 2).

### MRI outcomes

#### Lateral meniscus extrusion

Pre-operatively, LME in LMPR intact group (1.43 ± 1.17 mm) was significantly smaller than that in the other two groups (LMPR repair group: 2.66 ± 0.80 mm, *P* = 0.005; LMPR resected group: 2.66 ± 0.79 mm, *P* = 0.006). LME in LMPR repair group decreased to 1.44 ± 0.58 mm significantly post-operatively (*P* = 0.001), while no significant changes were observed in the other two groups (LMPR intact group: 1.67 ± 0.70 mm; LMPR resected group: 2.51 ± 0.68 mm). Post-operative LME of LMPR resected group was significantly greater among the three groups (LMPR intact group: *P* < 0.001; LMPR repair group: *P* < 0.001).

#### Anterior tibial subluxation

In regard of ATSLC, there were significant decreases after operations among the three groups. However, difference of post-operative ATSLC did not reach significant level in multiple comparison. In comparison of ATSMC, this figure was smaller in LMPR intact group (4.17 ± 1.29 mm) than LMPR repair group (6.03 ± 0.65 mm, *P* = 0.002) and LMPR resected group (7.10 ± 1.75 mm, *P* < 0.001). Significant reductions in ATSMC were detected in all the three groups after surgeries, which was similar to ATSLC. Likewise, we did not observe significant different in the post-operative subgroup analysis. In the respect of the difference between ATSLC and ATSMC, it progressed from 0.88 ± 1.57 mm pre-operatively to 3.11 ± 0.50 mm post-operatively with a significant difference (*P* = 0.006) in LMPR resected group. Additionally, post-operative ATSLC-ATSMC difference was significantly higher in LMPR resected group than that in LMPR intact group (0.96 ± 3.30 mm, *P* = 0.031) and LMPR repair group (1.01 ± 0.71 mm, *P* = 0.048).

#### Meniscus healing

**Fig. 6:**
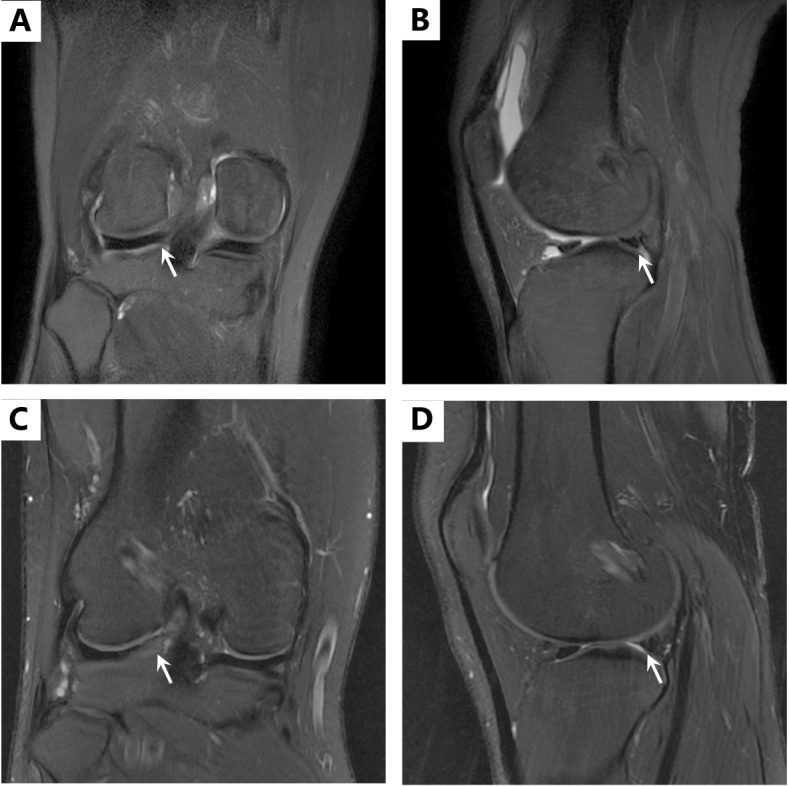
Healing status on magnetic resonance image 2 years after LMPR repair, white arrows showed the posterior root of lateral meniscus. **A**, **B** Complete healing: an identifiable meniscus on both sagittal and coronal scans. **C**, **D** Partial healing: an identifiable meniscus was showed but high signal could be detected among the normal dark meniscal signal on both sagittal and coronal scans (*LMPR* lateral meniscus posterior root)

Among all the 16 patients in LMPR repair group, 13 patients (81.2%) showed complete healing and 3 patients (18.8%) showed partial healing. No repeat tears were detected (Fig. 6).

#### Signal/noise quotient

**Fig. 7:**
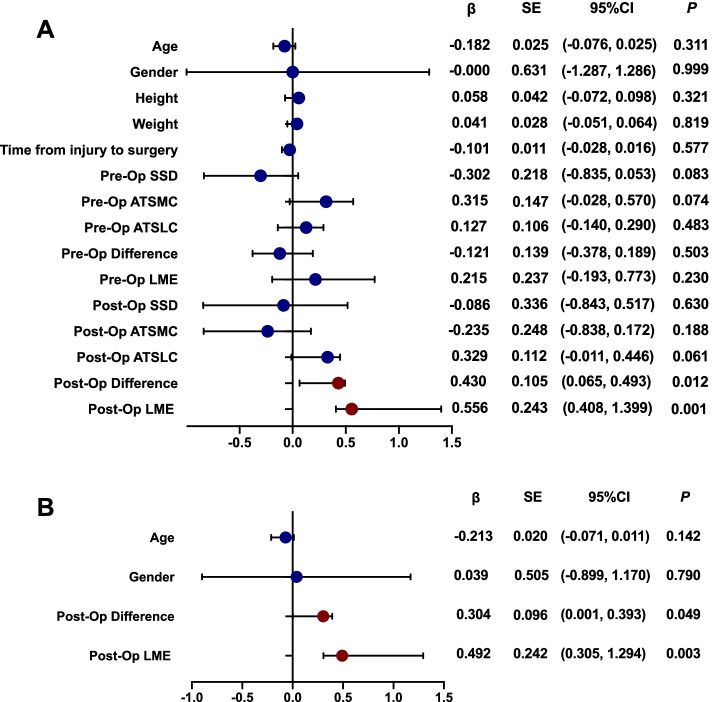
Univariate and multivariate linear regression analysis. The forest plot of β showed the correlation. (*SSD* side-to-side difference, *ATSMC* anterior tibial subluxation of medial compartment, *ATSLC* anterior tibial subluxation of lateral compartment, *LME* lateral meniscus extrusion, *SE* standard error, *CI* confidence interval)

At 2-year follow-up, the SNQ in LMPR resected group (4.48 ± 1.76) was significantly higher in LMPR intact group and LMPR repair group (2.73 ± 1.00, *P* = 0.004; 2.41 ± 1.20, *P* = 0.002, respectively; Table 3). We conducted a univariate regression analysis (Fig. 6) and found that post-operative ATSLC-ATSMC difference (β = 0.430, *P* = 0.012) and LME (β = 0.556,* P* = 0.001) were significantly associated with graft SNQ value in a total of 49 ACL grafts. The aforementioned 2 factors, with the addition of age and gender as known influencing factors in previous regression models, were included in a multivariate regression analysis. Post-operative ATSLC-ATSMC difference (β = 0.304, *P* = 0.049) and LME (β = 0.492, *P* = 0.003) also showed a significant association with graft SNQ value in the multivariate model (Fig. 7).

**Table 3:** MRI outcomes (mean ± SD) among LMPR intact, repair and resected group

Parameters	LMPR intact	LMPR repair	LMPR resected	*P* value		
				intact vs repair	repair vs resected	resected vs intact
ATSMC						
Pre-operative	4.17 ± 1.29	6.03 ± 0.65	7.01 ± 1.75	0.002	0.081	0.000
Post-operative	2.95 ± 1.35	3.15 ± 0.93	2.49 ± 0.67	0.668	0.196	0.323
* P* value	0.008	0.000	0.000	/	/	/
ATSLC						
Pre-operative	6.45 ± 2.94	8.17 ± 1.87	8.00 ± 2.42	0.110	0.876	0.164
Post-operative	3.91 ± 3.23	4.16 ± 1.23	5.60 ± 0.77	0.795	0.175	0.091
* P* value	0.007	0.000	0.007	/	/	/
ATSMC-ATSLC difference						
Pre-operative	2.22 ± 2.07	2.13 ± 2.00	0.88 ± 1.56	0.920	0.167	0.115
Post-operative	0.96 ± 3.30	1.01 ± 0.71	3.11 ± 0.50	0.955	0.048	0.031
* P* value	0.117	0.080	0.006	/	/	/
LME						
Pre-operative	1.43 ± 1.17	2.66 ± 0.80	2.66 ± 0.79	0.005	0.998	0.006
Post-operative	1.68 ± 0.70	1.44 ± 0.58	2.51 ± 0.67	0.273	< 0.001	< 0.001
* P* value	0.392	0.001	0.701	/	/	/
SNQ	2.74 ± 1.00	2.41 ± 1.20	4.48 ± 1.76	0.550	0.002	0.004

*MRI* magnetic resonance imaging, *SD* standard deviation, *LMPR* lateral meniscus posterior root, *ATSMC* anterior tibial subluxation of medial compartment, *ATSLC* anterior tibial subluxation of lateral compartment, *LME* lateral meniscal extrusion, *SNQ* signal/noise quotient

## Discussion

The principal results of our current retrospective study supported the initial hypotheses that successful transtibial repair of LMPR would reduce meniscal extrusion significantly and be beneficial for ACL graft maturation with regard of SNQ. Additionally, linear regression analysis demonstrated that the difference between anterior tibial subluxation of medial and lateral compartment and lateral meniscus extrusion would have significantly positive influence on the ACL graft’s SNQ at the end of follow-up. It is also corresponded to the favorable clinical evaluation that functional outcomes after LMPR repair concomitant with ACL reconstruction significantly improved post-operatively, which is in accordance with the latest systemic review ^21^. These promising results support the indication for suture repair of LMPRT.

Transtibial pull-out technique, which combined an all-inside repair of the meniscus root by self-retrieving suture passer and a transosseous repair utilizing a bone tunnel to shuttle sutures for eventual fixation of the sutures to the bone, is a popularized procedure for LMPRT [16]. It is considered beneficial for maintaining tibiofemoral contact mechanics. LaPrade [16] reported that transtibial repair of LMPRT significantly reduced mean contact pressures in a cadaveric study. In a finite element analysis, Wang [3] demonstrated that repair of LMPRT resulted in a significant decrease in either joint contact area or contact stress, which was comparable to that of a normal knee. Thus, the current literature has put a great emphasis on restoring the integrity of LMPR and its hoop tension so that it could act as a restraint to meniscal extrusion. In this study, we found that lateral meniscus extrusion in LMPR repair group was significantly reduced by approximately 1.2 mm than the figure pre-operatively, which was also smaller than that in LMPR resected group and comparable to LMPR intact group. These results confirmed the biomechanical advantages of transtibial repair. The healing rate of LMPR after transtibial procedure range from 48 to 78% based on second-look arthroscopic surgery [15]. In our current study, we used MRI to assess the healing status of LMPR and detected a complete healing rate of 81.2%. The promising healing rate might be attributed to the bone marrow bleeding introduced by additional tibial bone tunnel in the footprint region [22].

Another concern about LMPR is its importance as a stabilizer for the knee. ACL reconstruction is considered as the standard procedure for ACL deficient knee with the purpose of restoring the knee kinematics. Nonetheless, Almekinders [23] described the irreducible subluxation of the knee despite of successful ACL reconstruction. As was illustrated in biomechanical and clinical studies, posterior horn of meniscus played the wedge effect in maintaining the stability of the knee [8, 14]. Grassi [24] reported that the anterior tibial subluxation of lateral compartment in extension in patients with an ACL injury combined with lateral meniscal defect was increased by 1.38-fold when compared with those with an intact lateral meniscus. In a latest clinical study, Zheng [14] reported that concomitant LMPR lesions increase the anterior tibial subluxation of lateral compartment in ACL injured knee. In our current study, although the difference of anterior tibial subluxation of lateral compartment in unloaded state among LMPR intact, repair and resected group did not reach significant level, we demonstrated that the difference between anterior tibial subluxation of medial and lateral compartment was significantly lower in LMPR repair group than that in LMPR resected group, which was comparable to LMPR intact group. The effect of LMPR on anterior tibial subluxation of lateral compartment was consistent with other previously published reports. LMPR has been recognized as a critical secondary stabilizer for anterolateral rotational stability [25]. A recent cadaveric study performed by Tang [13] demonstrated that LMPR transtibial repair in combination with ACL reconstruction significantly reduced the anterior translation of tibial plateau in a simulated pivot-shift maneuver. Therefore, we regarded transtibial repair of LMPR worthy of recommendation based on it biomechanical advantages.

Combined meniscal lesions are common in patients with ACL injuries, so we investigated the effect of the repair of LMPR on the outcome of ACL reconstruction. SNQ is a widely accepted parameter that reflects maturation and biomechanical strength of ACL graft [18, 20]. In the present study, SNQ of ACL graft in LMPR repair group was lower than the figure in LMPR resected group at 2-year follow-up. We considered this in line with the biomechanical reports. Cadaveric study reported that repair of LMPR improved the ACL graft force close to that of the intact ACL under anterior tibial loading [13]. To add to that, the relative anterior translation of tibial tunnel subsequent to the post-operative tibial subluxation would furtherly lead to intercondylar notch impingement with ACL graft [26]. Thus, this altered kinematics would be detrimental for the graft. It was confirmed that stable biomechanical environment is beneficial for the maturation and ligamentization for ACL graft. Additionally, we detected that 2-year SNQ of ACL graft had a significant positive association with the difference between anterior tibial subluxation of medial and lateral compartment based on the regression analysis. We inferred this as another implication of the reduced of internal tibial rotation after the reconstruction of LMPR. With respect to either anterior tibial subluxation of medial or lateral compartment as a single variable, their correlations with SNQ were not significant. We attributed this to the satisfactory restoration of anterior–posterior stability after anatomical ACL reconstruction, which was also in line with the relatively low SNQ post-operatively when compared to that in the published reports [20]. Another positive association was detected between 2-year-SNQ and lateral meniscus extrusion. We suspected that as extrusion of lateral meniscus increased, the role of LMPR as the secondary stabilizer during pivot-shift trend would be less sufficient, and thus a greater stress would be imposed on ACL graft.

In the current study, the difference of MRI parameters was in line with clinical functional outcomes. As was discussed previously, LMPR repaired knees showed smaller difference between anterior tibial subluxation of medial and lateral compartment and lateral meniscus extrusion when comparing with LMPR resected knees. Therefore, LMPR repaired knees could provide stability in performing more exquisite activities and avoid “giving way” sign. We considered this the reason why patients with LMPR repaired knees scored higher in Lysholm system than those who had LMPR resected. The current literature [15, 16, 21] had proved the effectiveness of LMPR repair when compared with the pre-operative status, but our results confirmed the advantage of LMPR repair over partial meniscectomy in radiographic aspect. It was reported that patients with slight knee laxity could reach acceptable outcome, and a period progression of rehabilitation could lead to improvement in neuromuscular function [27, 28]. Since clinical scores may not be sensitive enough to elucidate outcome differences, and hence we considered this the reason why our series reached comparable IKDC and Tegner scores post-operatively.

This study had some limitations. First, as mentioned above, the number of patients included in the final analysis was small and a follow-up of 2 year was short. So, a larger-sized, long-term study is on the necessity in the future. Second, some patients in LMPR intact group claimed that they took more than 2 years after the first injury. Meanwhile, patients in LMPR intact group seldom had combined injury, which might make them take longer to experience specific symptoms of the injured knee. These may introduce recall and selection bias. Third, LMPRT could be divided into avulsion and radial tear aforementioned. However, due to the small sample size, we did not perform subgroup analysis with respect to these subtypes. Forth, we did not investigate the integrity of meniscofemoral ligament in this study. Although the correlation between knee stability and meniscofemoral ligament was still a debatable issue, its role on ACL graft needs to be clarify. Additionally, we used hamstring autografts as the graft choice in this study, so the effects of LMPR on other ACL grafts such as patellar tendon, quadriceps tendon were unclarified. Last but not least, the retrospective design of the current study made the patient selection a confounding factor.

## Conclusion

Transtibial repair of LMPR concomitant with ACL reconstruction restored translational stability, reduced meniscus extrusion, making it beneficial for ACL graft maturation.

## Data Availability

The data and materials used and/or analyzed during the current study are not publicly available but available from the corresponding author on reasonable request.

## Abbreviations

ACL: Anterior cruciate ligament
ATSLC: Anterior tibial subluxation of latreral compartment
ATSMC: Anterior tibial subluxation of medial compartment
IKDC: : International Knee Documentation Committee
LME: Lateral meniscus extrusion
LMPR: Lateral meniscus posterior root
LMPRT: Lateral meniscus posterior root tear
MRI: Magnetic resonance imaging
PCL: Posterior cruciate ligament
ROI: Regions of interest
SNQ: Signal/noise quotient
SSD: Side-to-side difference
